# Diversity Analysis of Fecal Microbiota in Goats Driven by White Blood Cell Count

**DOI:** 10.3390/microorganisms14010259

**Published:** 2026-01-22

**Authors:** Meng Zeng, Hanlin Zhou, Qun Wu, Ke Wang, Hu Liu, Yuanting Yang, Weishi Peng, Anmiao Chen, Xiaoyan Deng, Chihai Ji, Xiaosong Zhang, Jiancheng Han

**Affiliations:** 1 Zhanjiang Experimental Station, Chinese Academy of Tropical Agricultural Sciences, Zhanjiang 524013, China; zmeng0909@163.com (M.Z.); wuqun.2006@163.com (Q.W.); lp_wangke@163.com (K.W.); liuh2018@lzu.edu.cn (H.L.); ytyang10@163.com (Y.Y.); m17378095519@163.com (W.P.); cam1835287831@163.com (A.C.); 8376025735@163.com (X.D.); 2 Institute of Agricultural and Animal Husbandry Industry Development, College of Animal Science and Technology, Guangxi University, Nanning 530004, China; 3 South China Sea Fisheries Research Institute, Chinese Academy of Fishery Sciences, Guangzhou 510300, China; jichihai@163.com; 4 College of Veterinary Medicine, China Agricultural University, Beijing 100193, China; zhangxsgs@cau.edu.cn

**Keywords:** fecal microorganisms, white blood cell, Leizhou goat, blood parameters

## Abstract

The Leizhou goat is a vital indigenous breed, yet its disease resilience can complicate early health monitoring. The white blood cell (WBC) count is a key indicator of immune status, but its relationship with the gut microbiota remains uncharacterized in this breed. This study aimed to characterize the fecal microbiota of Hainan black goats stratified based on their WBC counts. The goats were stratified into Lower, Middle, and High WBC groups based on peripheral WBC counts to compare their fecal microbiota and identify potential associations with systemic immunity. Significant differences in microbial alpha- and be-ta-diversity were observed among the groups, with the High WBC group showing the greatest richness. The microbiota was dominated by *Bacillota* and *Bacteroidota* at the phylum level. Linear discriminant analysis Effect Size (LEfSe) identified specific taxa en-riched in each group, such as *Ruminococcusin* the High WBC group. Critically, Spearman correlation analysis demonstrated significant positive correlations between WBC counts and the relative abundance of genera like *unclassified_f_Oscillospiraceae* and *unclassi-fied_c_Clostridia*. These findings demonstrate that WBC counts are significantly associated with distinct shifts in the gut microbial community structure of Hainan black goats. The identified WBC-associated microbial biomarkers suggest a link between the gut microbi-ome and host immune regulation, providing a foundation for future research on microbi-ota-mediated health assessment in goats.

## 1. Introduction

The Leizhou goat, also referred to as the Hainan black goat, is a recognized indigenous breed in China, valued for its high fecundity and productivity [1]. Adult does typically weigh 20.0–28.6 kg and exhibit efficient growth performance under tropical conditions, with reported average daily gains of 140 g/day during the fattening period [2]. Their notable heat tolerance is evidenced by stable physiological parameters (e.g., rectal temperature) under high temperature-humidity index conditions [2,3].This breed exhibits strong adaptability to tropical and subtropical climates, characterized by robust constitutions and considerable disease resistance [4]. However, this same resilience can lead to subtle or absent clinical presentations in the early stages of disease, making early detection challenging [5]. Often, by the time overt symptoms become apparent, the condition has significantly progressed, frequently resulting in diminished treatment efficacy [4]. Consequently, it is imperative to implement consistent health monitoring and adhere to a preventative healthcare strategy in daily breeding management [6]. Blood parameters serve as indicators of an animal’s physiological state and health, and are influenced by nutritional status [7,8,9]. Hematological evaluation also facilitates disease diagnosis and the early detection of abnormalities caused by infections or environmental stressors [6,10]. However, hematological variation may be influenced by factors such as breed, age, nutritional status, physiological condition, altitude, season, reproductive stage, and management practices [11,12]. Although Hainan black goats hold substantial economic and nutritional value in the coastal areas of southern China, there remains a notable lack of scientific research focused on their fundamental physiological and hematological traits.

The total white blood cell (WBC) count is a fundamental, routinely measured hematological parameter. It is valued as an early and low-cost indicator of physiological stress, systemic inflammation, and subclinical disease—even within the established normal range [13]. Beyond this diagnostic utility, WBCs—particularly the predominant neutrophil subset—are active participants in immune-microbiota crosstalk [14]. For instance, neutrophils help shape the gut microbial environment through mechanisms including the secretion of antimicrobial peptides [15,16]. Conversely, the intestinal microbiota is a critical modulator of host immunity, producing metabolites and signaling molecules that influence systemic inflammatory tone and hematopoiesis [17]. This is evidenced by studies showing that intestinal microbiota depletion impairs the recovery of lymphocyte and neutrophil counts [18]. Given this established bidirectional relationship, we hypothesized that variation in peripheral WBC counts—a readily accessible measure of immune status—could serve as a proxy for underlying shifts in the gut microbial ecosystem. However, while the microbiota’s influence on leukocytes is recognized, the potential for using routine hematological parameters like WBC profiles to infer gut microbiota composition in livestock—particularly in resilient breeds such as the Leizhou goat—remains underexplored.

Recent research has expanded our understanding of the gut microbiota’s role from regulating local mucosal immunity to influencing systemic immunoregulation and, critically, hematopoiesis [19]. Beyond modulating mucosal immunity, commensal microbes are now recognized as key regulators of systemic immunity, influencing the development and maintenance of innate immune cells—including neutrophils, monocytes, and macrophages—at distant sites [20]. Under homeostatic conditions, the gut microbiota actively supports the maintenance of both hematopoietic stem cell- and embryo-derived myeloid populations [20]. For example, basal microbial stimulation in conventional mice regulates granulopoiesis, promoting the generation of a reserve pool of myeloid cells within the bone marrow [19,20,21]. Collectively, this evidence positions the gut microbiota as a fundamental regulator of systemic immunity via the modulation of hematopoiesis.

Building on this mechanistic insight, we hypothesize that shifts in the intestinal microbial community could, via these hematopoietic pathways, directly affect the abundance and composition of peripheral immune cells—including the readily measurable WBC count. Our study therefore aimed to translate this mechanistic knowledge into a practical application by investigating whether routine hematological parameters, specifically WBC profiles, can serve as accessible peripheral indicators of gut microbiota composition in a livestock model—the Leizhou goat.

A well-balanced intestinal microbiota is beneficial to the host [22]. The continuous interaction between the intestinal microbiota and the host’s immune system significantly influences the induction, regulation, and suppression of local and systemic immune responses [14,23]. The immune system maintains intestinal tissue homeostasis by remaining vigilant against invasive microorganisms while restraining excessive inflammatory responses toward the commensal microbiota [24]. Thus, against the backdrop of genetic susceptibility and environmental factors, alterations in microbiota composition are potentially important as modulators and triggers of disease [25]. Intestinal dysbiosis involves the overgrowth of pathogenic bacteria and increased secretion of bacterial products, such as lipopolysaccharides, peptidoglycans, DNA, and outer membrane proteins, into the circulatory system, resulting in chronic immune activation [25,26].

Given the numerous connections between the intestinal microbiota and host health, it is particularly important to analyze the relationship between microbial changes and the occurrence, progression, and prognosis of diseases. The identification of pathogenic intestinal microorganisms associated with phenotypes and diseases through fecal microbiome analysis is a commonly used method for studying the intestinal microbiome [27]. Feces are commonly used as a proxy for the intestinal microbiota due to their accessibility and non-invasive nature [27]. The collection of appropriate microbiota samples is crucial. Fecal material that is instantly frozen at −80 °C and maintains microbial integrity without preservatives is widely regarded as the gold standard for intestinal microbiota analysis. This approach preserves microbial components comparable to those in fresh samples and avoids potential preservative effects [28].

We hypothesize that variation in peripheral WBC levels is influenced by shifts in the composition and function of the intestinal microbiome, which are in turn linked to alterations in immune regulation. Therefore, this study aimed to: (1) compare the differences in fecal microbial α-diversity (e.g., Shannon index) and β-diversity (e.g., Bray–Curtis dissimilarity) among goats stratified into high, middle, and low WBC ratio groups; and (2) identify specific bacterial taxa (at the genus or species level) whose relative abundances correlate significantly with key hematological parameters, including white blood cell count (WBC), lymphocyte percentage (LYM%), and granulocyte absolute count (GRAN#). Through these analyses, we sought to delineate associations between peripheral immune status (reflected by WBC profiles) and the gut microbial ecosystem in Leizhou goats.

## 2. Materials and Methods

### 2.1. Animal Ethics

The animal study protocol was approved by the Animal Ethics Committee of the Zhanjiang Experimental Station, Chinese Academy of Tropical Agricultural Sciences (Approval No.: CATAS-20240004ZES).

### 2.2. Experimental Design, Animals, and Diet

The experiment was conducted at a goat farm, located in Zhanjiang, Guangdong Province, China, owned by the Zhanjiang Experimental Station, Chinese Academy of Tropical Agricultural Sciences. A cohort of clinically healthy female Leizhou goats (aged 12 ± 2 months, mean ± SD) was initially screened to ensure a uniform baseline health status. The screening criteria included: (1) no clinical signs of disease (e.g., normal appetite, posture, and fecal consistency; no signs of lameness, coughing, or nasal discharge) upon veterinary examination; (2) rectal temperature, heart rate, and respiration rate within normal physiological ranges for the breed and age; and (3) no history of major illness in the preceding two months. Furthermore, pregnant goats were excluded from the study. From this rigorously screened cohort, a final group of 27 goats that also exhibited similar body condition (body weight: 18.74 ± 1.03 kg) was established.

This final cohort of 27 animals was used to investigate the association between blood parameters and the gut microbiome. Notably, all samples were collected sequentially on the final day of the trial, with blood sampling preceding fecal collection.

Following a 15-day adaptation period, the formal experiment was conducted over a 60-day period from 15 May to 15 July 2024. Throughout this period, all goats were maintained under uniform management conditions to minimize potential environmental confounding factors and ensure the validity of the results. They were fed twice daily (08:00 and 16:30) with a diet formulated according to the Chinese national standard “Nutrient Requirements of Meat-Type Sheep and Goat (NY/T 816-2021)” [29] (Table 1), with water available ad libitum. Animals were maintained under an intensive stall-feeding system in a single pen (1.5 m^2^/animal) [30]. The controlled environmental conditions (25–28 °C, 50–60% relative humidity, natural light) yielded an average temperature-humidity index (THI) of 68.6–73.4. For the Leizhou goat breed, which exhibits notable heat tolerance, this range is non-stressful. This is corroborated by studies showing that even at a higher THI (78.07) [31], these goats do not increase behaviors like lying down and drinking to dissipate heat, consistent with known breed-specific variations in thermal comfort [32,33].

### 2.3. Fecal Sample Collection

Fecal samples were collected directly from the rectum of each goat prior to morning feeding using sterile gloves. Approximately 5 g of fecal matter was collected from each animal to ensure a sufficient quantity for analysis. Immediately upon collection, the samples were transferred into sterile tubes, snap-frozen in liquid nitrogen, and stored at −80 °C until DNA extraction. All procedures were performed rapidly by trained personnel to minimize stress to the animals.

### 2.4. Blood Sample Analysis

Blood samples (approximately 5 mL each) were collected via jugular vein puncture using sterile disposable syringes. Immediately after collection, the samples were transferred into EDTA-coated anticoagulant tubes, gently inverted 5–8 times to ensure thorough mixing with the anticoagulant, and stored at 4 °C for transport to the laboratory. Hematological analyses were performed within 2 h of sample collection using an automated hematology analyzer (scil Vet abc Hematology Analyzer, scil Animal Care Company, Altorf, France) following the manufacturer’s standardized operating procedures. A full hematological panel was performed, measuring parameters including white blood cell count (WBC), lymphocyte percentage (LYM%), mid-range cell percentage (MID%), granulocyte percentage (GRAN%), granulocyte absolute count (GRAN#), red blood cell count (RBC), hematocrit (HCT), mean corpuscular volume (MCV), and mean corpuscular hemoglobin concentration (MCHC). Among these, the WBC count served as the primary variable of interest for grouping the animals and investigating its association with the gut microbiota.

The total WBC count, a key hematological indicator of physiological and immune status [13], was measured in these goats to facilitate a comparative analysis of the fecal microbiota across a spectrum of WBC values (i.e., high, medium, and low ranges). Reference data on hematological parameters in Leizhou goats are limited. Available studies report mean baseline WBC counts of 14.54 × 10^9^/L [3] and 16.21 ± 2.56 × 10^9^/L [35] in control groups, with values decreasing under experimental conditions such as nano-selenium supplementation or heat stress. Based on this existing data range, the 27 goats in this study were stratified into three groups for comparative analysis: a Lower group (WBC count < 15.00 × 10^9^/L), a Middle group (WBC count ≥ 15.00 and < 20.00 × 10^9^/L), and a High group (WBC count ≥ 20.00 × 10^9^/L).

### 2.5. Fecal Microbiota Analysis

Total genomic DNA was extracted from 1.00 g of each fecal sample using a commercial DNA extraction kit (DP328, Tiangen Biotech, Beijing, China). The quality and concentration of the extracted DNA were assessed using a Thermo Scientific NanoDrop 2000 spectrophotometer (Thermo Fisher Scientific, Waltham, MA, USA) and by 1% agarose gel electrophoresis. The hypervariable V3–V4 regions of the bacterial 16S rRNA gene were amplified using the primers 338F (5′-ACTCCTACGGGAGGCAGCAG-3′) and 806R (5′-GGACTACHVGGGTWTCTAAT-3′) [36]. The PCR amplification was carried out under the following conditions: initial denaturation at 95 °C for 3 min; 30 cycles of denaturation at 95 °C for 30 s, annealing at 55 °C for 30 s, and extension at 72 °C for 45 s; and a final extension at 72 °C for 10 min, followed by holding at 10 °C. The PCR reactions were set up in triplicate with a total volume of 20 µL per reaction. Each reaction mixture contained 10 µL of 2× Pro Taq master mix, 0.8 µL of each primer (5 µM), 10 ng of template DNA, and were brought to the final volume with ddH2O. The success of the PCR amplification was verified by 2.0% agarose gel electrophoresis (Axygen Biosciences, Union City, CA, USA). Subsequent steps, including quality filtering, clustering, and analysis of the 16S rRNA sequencing data, were performed according to the method described by Liu et al. (2024) [36]. The PCR amplicons were purified, quantified, and pooled in equimolar amounts for paired-end sequencing on an Illumina MiSeq PE300 platform (Illumina, San Diego, CA, USA) by Majorbio Bio-Pharm Technology Co., Ltd. (Shanghai, China).

### 2.6. Statistical Analysis

Hematological parameters were analyzed using IBM SPSS Statistics (version 27; IBM Corp., Armonk, NY, USA). Quantitative data are expressed as the mean ± SD. The goats were stratified into three groups (Lower, Middle, High) based on the natural distribution of their baseline WBC counts, resulting in unequal group sizes (n = 13, 6, and 8, respectively).

Additional statistical analyses were conducted using SAS (version 9.4; SAS Institute Inc., Cary, NC, USA), and figures were generated using R software (version 4.2.2; R Foundation for Statistical Computing, Vienna, Austria). The Shapiro–Wilk test was employed to assess the normality of data distribution. Differences in α-diversity indices among the three WBC groups were evaluated using the Kruskal–Wallis H test, with post hoc pairwise comparisons performed using the Benjamini–Hochberg method to control the false discovery rate (FDR). Beta-diversity differences were assessed by permutational multivariate analysis of variance (PERMANOVA) based on Bray–Curtis distances with 999 permutations. Spearman’s rank correlation analysis was used to examine relationships between microbial relative abundances and hematological parameters, with *p*-values adjusted for multiple comparisons using the FDR. For all analyses, a two-tailed *p*-value of less than 0.05 (*p* < 0.05) was considered statistically significant, unless otherwise noted.

## 3. Results

### 3.1. Differences in Hematological Parameters Among Goat Groups Defined by Initial WBC Levels

Table 2 presents the measured hematological parameters of experimental goats categorized into three groups (Lower, Middle, High) based on their initial WBC counts. The groups comprised different numbers of animals (n = 13, 6, and 8, respectively), and a total of nine key blood indicators were measured, including WBC, LYM%, RBC, and others. The data clearly shows variations in the values of multiple parameters across the different groups, thereby laying the data foundation for the subsequent analysis of the associations between WBC levels and indicators such as the intestinal microbiota.

### 3.2. Sequencing Data

**Figure 1:**
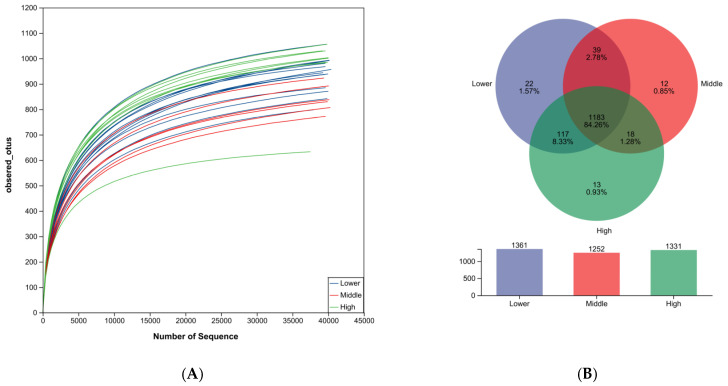
(**A**) Rarefaction curves illustrating the number of observed OTUs as a function of sequencing depth for each sample. The plateau reached by all curves indicates that the sequencing depth was adequate to capture the majority of microbial diversity present in the samples. (**B**) Venn diagram depicting the distribution of unique and shared OTUs among the three experimental groups (Lower, Middle, and High).

Following quality control, a total of 11,697,213 sequences were obtained from the samples. The average number of sequences per sample was 8330. The sequencing quality was assessed using rarefaction curves. All rarefaction curves reached a plateau, indicating that the number of operational taxonomic units (OTUs) approached saturation (Figure 1A). The rarefaction curves indicated that the sequencing depth was sufficient to capture the majority of the microbial diversity. A total of 1404 OTUs were identified. Among these, the Lower and Middle groups shared 1222 OTUs. The Lower and Middle groups contained 22 and 12 unique OTUs, respectively. Similarly, the Middle and High groups shared 1201 OTUs, with 12 and 13 unique OTUs, respectively (Figure 1B).

### 3.3. OUT Abundance Analysis

**Figure 2:**
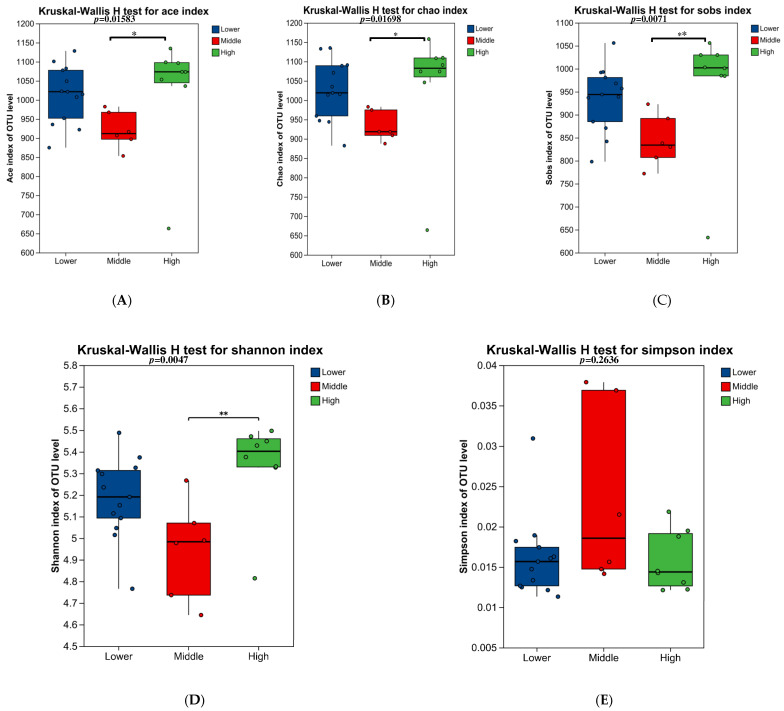
Assessment of the α-diversity of the fecal microbiota across the three experimental groups. (**A**) Ace, (**B**) Chao, (**C**) Sobs, (**D**) Shannon, and (**E**) Simpson indices. Bars represent the mean values of the three groups, and error bars indicate the standard error of the mean (SEM). *p* < 0.05 was considered statistically significant. * indicates FDR < 0.05, and ** indicates FDR < 0.01 compared with another group.

The α-diversity of the microbial communities was assessed using the Ace, Chao, Sobs, Shannon, and Simpson indices (Figure 2). The Sobs index reflects the observed species richness. The Chao and Ace indices are estimators of species richness, particularly for rare species. The Shannon index incorporates both species richness and evenness to represent community diversity.

In this study, significant differences in community richness were observed among the groups. Specifically, the Sobs, Chao, and Ace indices, all indicators of richness, were significantly lower in the Middle group compared to the High group (*p* < 0.05), but showed no significant differences between the Lower group and the other two groups (Figure 2A–C). Similarly, the Shannon index was also significantly lower in the Middle group than in the High group (Figure 2D). In contrast, the Simpson index, reflecting community evenness, showed no significant differences among the three groups (Figure 2E).

**Figure 3:**
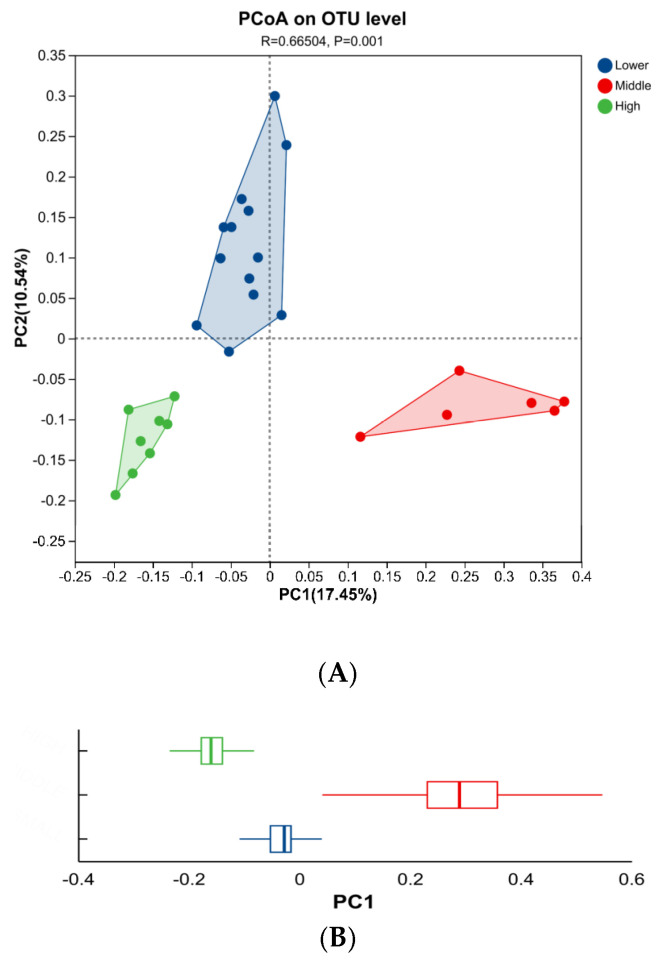
Beta diversity analysis of the intestinal microbiota among the three experimental groups. (**A**) PCoA plot based on the Bray–Curtis distance matrix, illustrating the overall structural differences in microbial communities. The spatial distribution of samples indicates a clear separation between the groups. (**B**) Box plot depicting the dispersion of samples from each group along the first PC1 axis. The box represents the interquartile range (IQR), the horizontal line within the box marks the median, and the whiskers extend to 1.5 times the IQR.

Significant differences in the β-diversity of microbial community composition were observed among the Lower, Middle, and High groups, as visually represented in Figure 3. Panel A shows the results of principal coordinates analysis (PCoA), an unconstrained ordination method. Panel B presents a box plot depicting the dispersion of the sample groups along the principal coordinate 1 (PC1) axis. Comparative analysis of microbial community diversity revealed distinct compositional differences in the intestinal microbiota among the three experimental groups.

### 3.4. Community-Composition Analysis

**Figure 4:**
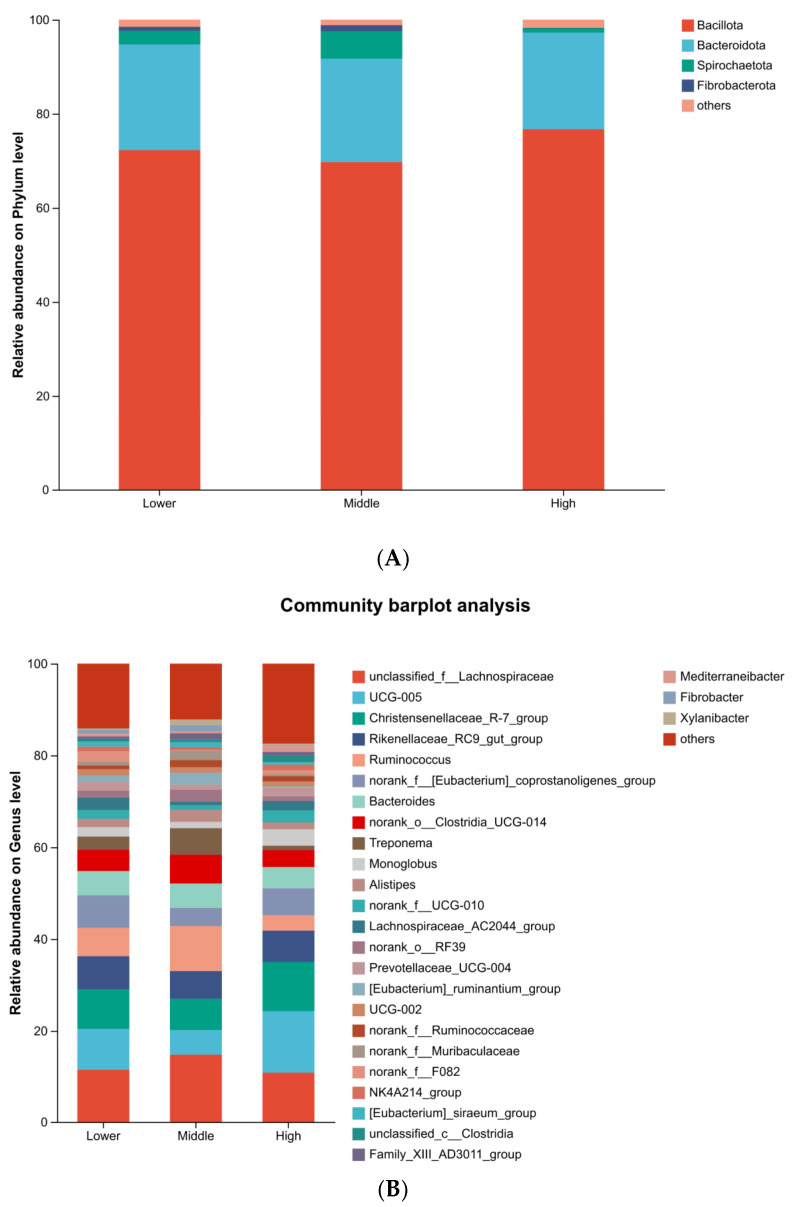
Comparative analysis of intestinal microbiota composition in goats with varying WBC ratios at the phylum (**A**) and genus (**B**) levels. Stacked bar charts illustrating the relative abundance (%) of the predominant bacterial taxa in the fecal microbiota of goats from the Low, Middle, and High WBC ratio groups.

The microbial composition of 27 samples was analyzed, and their average relative abundance at the phylum level was calculated. The core phyla included *Bacillota* (72.92%), *Bacteroidota* (21.64%), *Spirochaetota* (3.22%), and *Fibrobacterota* (0.73%), which accounted for 98.51% of the microbial composition across all samples (Figure 4A). Additionally, the core genera included *Lachnospiraceae_unclassified* (12.36%), *UCG-005* (9.26%), *Christensenellaceae_R-7_group* (8.72%), *Rikenellaceae_RC9_gut_group* (6.69%), *Ruminococcus* (6.44%), *norank_f_coprostanoligenes_group* (5.63%) and *Bacteroides* (5.09%), which collectively accounted for 54.19% of the identified microbial composition in all samples (Figure 4B).

At the phylum level, *Bacillota* (72.27%), *Bacteroidota* (22.46%), and *Spirochaetota* (29.24%) were the dominant taxa (>1%) in Group Lower. *Bacillota* (69.75%), *Bacteroidota* (21.96%), *Spirochaetota* (57.86%), and *Fibrobacterota* (1.33%) were the dominant taxa (>1%) in Group Middle. *Bacillota* (76.74%), *Bacteroidota* (20.51%), and *Spirochaetota* (1.73%) were the dominant taxa (>1%) in High Group.

At the genus level, the composition of the predominant taxa (relative abundance >1%) varied across groups. In the Lower group, the dominant genera were *Lachnospiraceae_unclassified* (11.47%), *UCG-005* (8.94%), *Christensenellaceae_R-7_group* (8.65%), *Rikenellaceae_RC9_gut_group* (7.20%), *Ruminococcus* (6.18%), *norank_f_coprostanoligenes_group* (7.09%), *Bacteroides* (5.30%), *norank_o_Clostridia_UCG-014* (4.62%), among others. In the Middle group, the community was characterized by a high abundance of *Lachnospiraceae_unclassified* (14.77%), *Ruminococcus* (9.81%), *norank_o_Clostridia_UCG-014* (6.27%), and Treponema (5.78%). In the High group, the predominant taxa included *UCG*-*005* (13.43%), *Christensenellaceae_R-7_group* (10.72%), *Lachnospiraceae_unclassified* (10.85%), and *norank_f_coprostanoligenes_group* (5.88%). The complete list of all predominant taxa for each group is provided in Supplementary Table S1.

### 3.5. LEfSe Analysis

**Figure 5:**
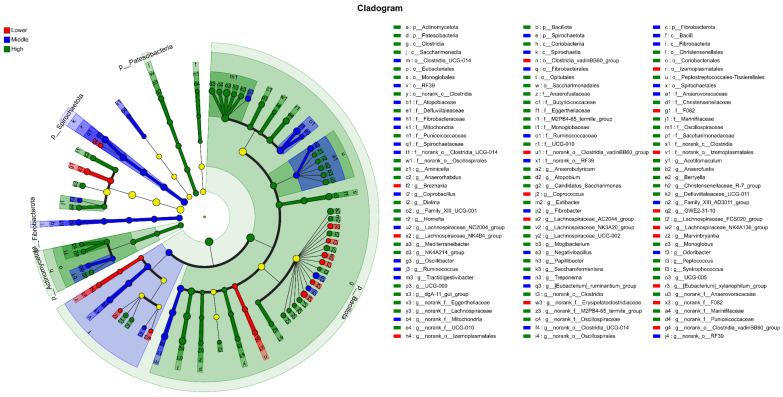
Cladogram generated from LEfSe analysis of the 16S rRNA gene sequencing data. The cladogram depicts the phylogenetic distribution of microbial taxa that were significantly enriched in each of the three groups. Colored nodes (blue, green, red) represent taxa identified as biomarkers for Group Lower, Middle, and High, respectively, with an LDA score greater than 2.0. Yellow nodes represent taxa that did not show significant differential abundance. The concentric circles represent the phylogenetic hierarchy from the phylum level (innermost circle) to the genus level (outermost circle).

Figure 5 presents the cladogram generated from the LEfSe analysis, illustrating the phylogenetic distribution of microbial taxa that are statistically significant biomarkers among the three groups (Lower, Middle, and High). The concentric circles represent taxonomic levels from the phylum (innermost circle) to the genus (outermost circle). Each node represents a specific taxon, and its diameter is proportional to the taxon’s relative abundance.

**Figure 6:**
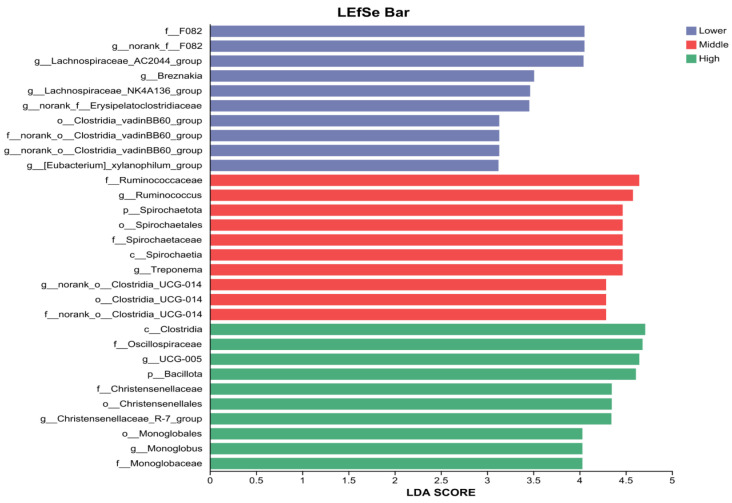
Linear Discriminant Analysis (LDA) Effect Size (LEfSe) histogram of differentially abundant bacterial taxa among the three experimental groups. The bar chart displays taxa with an LDA score greater than a predefined threshold (LDA > 2.0), indicating significant enrichment in a particular group. The length of each bar corresponds to the LDA score, which represents the effect size or magnitude of the difference contributed by that taxon. The color of the bars corresponds to the group (Lower, Middle, or High) in which the taxon was found to be significantly more abundant. Lower, low WBC group (n = 13); Middle, moderate WBC group (n = 6); High, high WBC group (n = 8).

According to the standard interpretation of LEfSe cladograms, nodes colored in blue, green, and red indicate taxa that were identified as key discriminators (biomarkers) for Group Lower, Group Middle, and Group High, respectively. Taxa colored yellow did not show statistically significant differences among the groups. The Linear Discriminant Analysis (LDA) score (Figure 6) associated with each colored taxon quantifies the effect size, or the degree of its influence, in distinguishing the groups, with a higher LDA score indicating a greater contribution to the differences observed in the microbial community structure.

LEfSe analysis further identified specific bacterial taxa that were significantly enriched in each group (LDA score > 2.0). At the phylum level, *Spirochaetota* was a distinctive biomarker for the Middle group, while *Bacillota* was significantly enriched in the High group. Significant differences were more pronounced at the genus level. The Lower group was characterized by an enrichment of taxa including *norank_f_F082*, *Lachnospiraceae_AC2044_group*, *Breznakia*, *Lachnospiraceae_NK4A136_group*, *norank_f_Erysipelatoclostridiaceae_group0*, *norank_o_Clostridia_vadinBB60_group*, and *[Eubacterium]_xylanophilum_group*. In the High group, a greater number of genera were enriched, with *Ruminococcus* and *Treponema* showing significant abundance, alongside *norank_o_Clostridia_UCG-014*, *UCG-005*, *Christensenellaceae_R-7_group*, and *Monoglobus*.

### 3.6. Strong Correlations Between WBC Levels and Specific Microbial Groups

**Figure 7:**
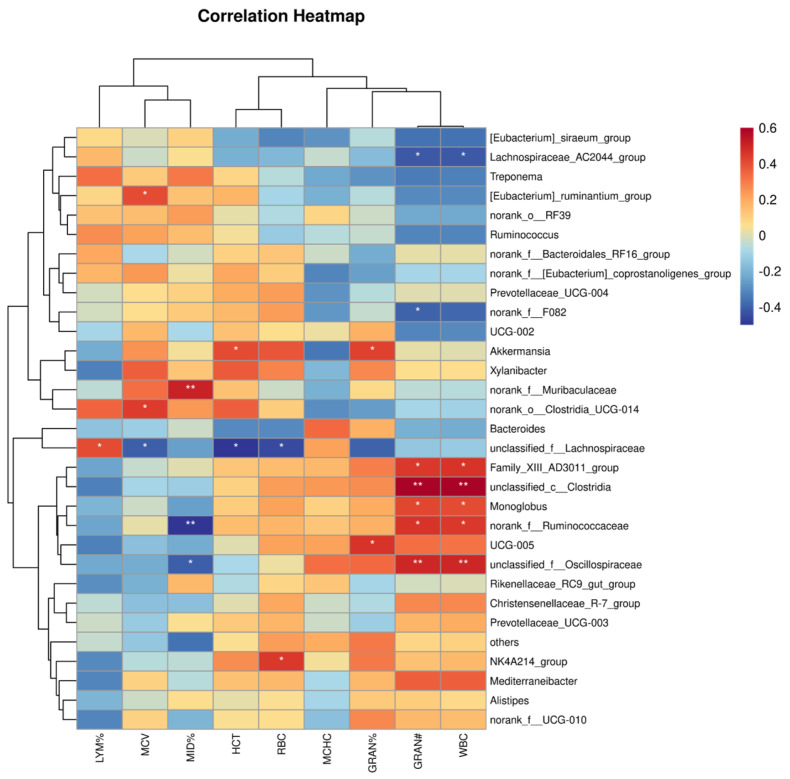
Correlation analysis between the relative abundance of the top 50 dominant microbial genera and clinical parameters. The heatmap depicts Spearman’s rank correlation coefficients, with color intensity and hue representing the strength and direction of the correlations (e.g., blue shades for positive correlations, red shades for negative correlations). Statistical significance levels are denoted by asterisks: * FDR < 0.05, ** FDR < 0.01.

Correlation analysis between the top 50 dominant microorganisms and a range of blood parameters is a powerful method for identifying microbial species with strong associations to host physiological indicators. As shown in Figure 7, the correlation heatmap illustrates Spearman’s rank correlations between the relative abundance of key microbial genera and hematological parameters, highlighting specific taxa with significant positive or negative links to WBC and other blood traits. These specific microorganisms are considered key flora due to their potential role in regulating health status or influencing blood parameters, thereby providing a viable approach for screening potential biomarkers to distinguish disease from health from a microbial perspective.

#### 3.6.1. Correlation of WBC Levels with Microbiota

A significant positive correlation was identified between WBC levels and the relative abundance of *Family_XIII_AD3011_group*, *Monoglobus*, and *norank_f_Ruminococcaceae* (all FDR < 0.05), as well as with *unclassified_f_Oscillospiraceae* and *unclassified_c_Clostridia* (FDR < 0.01).

#### 3.6.2. Correlation of Hematological Parameters with Microbiota

Analysis of the correlations between hematological parameters and intestinal microbiota composition revealed several significant associations. The percentages of LYM% and GRAN% demonstrated positive correlations with the relative abundance of certain intestinal microbial taxa. Conversely, HCT and the percentage of MID%—a composite parameter in leukocyte differential counts that primarily includes eosinophils, basophils, and monocytes—showed significant negative correlations with microbial abundance. These findings enhance our understanding of the potential mechanisms by which the intestinal microbiota modulates the host’s immune response to immunogens, particularly by highlighting its association with the proportional shifts in key leukocyte subsets.

#### 3.6.3. Indicators Without Statistical Significance

No statistically significant correlation was observed between the MCHC and the relative abundance of the various bacterial taxa analyzed in this study.

#### 3.6.4. Indicators with Strong and Significant Correlation

WBC count demonstrated strong positive correlations with the relative abundance of *unclassified_f_Oscillospiraceae* and *unclassified_c_Clostridia* (FDR < 0.01). Similarly, GRAN# was also strongly correlated with these same taxa. Furthermore, the percentage of MID% exhibited significant correlations with *norank_f_Muribaculaceae* and *norank_f_Ruminococcaceae* (FDR < 0.01).

In Hainan black goats, *Lachnospiraceae_AC2044_group* displayed a significant negative correlation with WBC counts, whereas several other microbial taxa showed significant positive correlations. Specifically, *Family_XIII_AD3011_group*, *Monoglobus*, and *norank_f_Ruminococcaceae* (FDR < 0.05), as well as *unclassified_f_Oscillospiraceae* and *unclassified_c_Clostridia* (FDR < 0.01). Furthermore, LYM% and GRAN% were also significantly positively correlated with the overall structure of the gut microbiota. Additional routine blood parameters, including MCV, MID%, HCT, RBC, and GRAN#, exhibited distinct correlation patterns, being positively correlated with certain intestinal microbes and negatively correlated with others.

## 4. Discussion

Owing to their strong adaptability and low maintenance requirements, goats have become a preferred livestock species worldwide. China is a leading global producer of goat meat, with an annual output of approximately 2.5 million tons. This output represents 43.8% of the Asian total and 33.5% of the global total. The Leizhou goat, the only indigenous breed in the Leizhou Peninsula and Hainan Island, exhibits strong adaptability to tropical and subtropical climates, along with robust constitution and high disease resistance [2]. However, these advantageous traits, particularly their resilience, may obscure the critical role of the gut microbiota in maintaining immune homeostasis. Given the established bidirectional crosstalk between the gut microbiota and systemic immunity—where the microbiota influences hematopoiesis and immune cell dynamics, and immune cells like neutrophils shape the microbial niche—we postulated that readily measurable peripheral immune markers, such as WBC counts, could reflect underlying shifts in the gut ecosystem [13,17,18]. Therefore, this study employed 16S rRNA gene sequencing to investigate the intestinal microbiota of Leizhou goats with varying WBC levels.

Rarefaction curves are used to assess the adequacy of sequencing depth and indirectly reflect the species richness in the samples. A plateau in the curves suggests that the sequencing depth is approaching saturation [37], indicating satisfactory sample uniformity and that the sampling effort is sufficient for reliable data analysis [38,39].

Alpha diversity was analyzed to evaluate the microbial species diversity within each sample. Significant differences (*p* < 0.05) in the Ace, Chao, Sobs, and Shannon indices were observed among the groups with lower (Low), intermediate (Middle), and higher (High) WBC levels. Notably, the group with the highest white blood cell levels (High) exhibited the highest alpha diversity indices, suggesting the greatest microbial species richness and diversity among all groups. Consistent with these findings, the Venn diagram revealed that the High group contained the highest number of OTUs (Figure 1A), a phenomenon potentially associated with the host’s physiological and health status [40].

High-throughput 16S rRNA gene sequencing was utilized to characterize the intestinal microbiota of Hainan black goats, bypassing the need for cultivation [41]. The analysis revealed that the intestinal microbiota was predominantly composed of *Bacillota*, *Bacteroidota*, *Spirochaetota*, and *Fibrobacterota*, which collectively accounted for 98.51% of the total microbial community. This microbial profile is consistent with those previously reported in fecal samples from goats [36], cattle [42], and dairy cows [43]. Although dominant phyla were consistent across groups, their relative abundances varied, suggesting that while the core fecal microbial community in goats is stable, WBC count fluctuations may substantially influence the proportional representation of these dominant taxa.

Twenty-seven dominant genera were identified. *Unclassified_f_Lachnospiraceae* was the most abundant, consistent with prior reports in Leizhou goats where it and *Oscillospiraceae_UCG-005* were predominant. In our study, *unclassified_f_Lachnospiraceae* was most abundant in the Middle WBC group. The designations *unclassified_c_Clostridia* and *unclassified_f_Lachnospiraceae* refer to incompletely classified taxa known to exert various health effects. Notably, *Lachnospiraceae* has been suggested to function as probiotics, enhancing fermentation efficiency and promoting host health [44].

Our findings align with the recognized intimate association between the intestinal microbiota and host immunity [14,27]. For instance, variations in WBC counts are known to correlate with shifts in microbial community structure [18], potentially explaining the differential abundance of *unclassified_f_Lachnospiraceae* observed here. *Ruminococcus*, involved in decomposing cellulose and hemicellulose to produce metabolites like acetic and butyric acid, contributes to ruminal stability as shown in yaks [45]. Its highest abundance in the Middle WBC group in this study underscores the complexity of microbiota-immune interactions in maintaining ecosystem stability and modulating immune responses [14].

A correlation was observed between the relative abundance of the phylum *Spirochaetotain* the gut microbiota and peripheral WBC counts. Its abundance exhibited a non-linear relationship with WBC counts: it increased significantly as counts rose from low to moderate, but then decreased markedly at high WBC levels. This pattern may be linked to immune regulatory processes within the host. Furthermore, shifts in the abundance of specific genera (e.g., *[Eubacterium]_siraeum_group*, *Ruminococcus*, and *Treponema*) suggest a potential bidirectional interaction: the gut microbiota may influence peripheral WBC counts, possibly via hematopoietic regulation [18], while changes in WBC levels may in turn reshape the microbial community structure [46].

Specifically, the relative abundances of *unclassified_c_Clostridia* and *unclassified_f_Oscillospiraceae* were positively correlated with WBC levels, whereas that of the *Lachnospiraceae_AC2044_group* was negatively correlated. These correlations imply that these microbial taxa may be involved in immunomodulation and could serve as potential indicators of host health status. Collectively, these findings provide new insights into the suboptimal microbiome and highlight potential targets for developing novel preventive and therapeutic strategies. Future research should focus on elucidating the causal relationships between the gut microbiota and host health, and evaluate the efficacy of microbiota-targeted interventions for immune system impairments.

Viewed from an ecological perspective, alterations in the intestinal microbiota may signify disruptions to ecosystem stability and function, potentially compromising the host’s ability to maintain homeostasis. These alterations may encompass disruptions in the production of essential metabolites, compromised intestinal barrier integrity, and dysregulated development and function of immune cells [47]. Environmental factors, particularly diet, are established as key determinants of intestinal microbiota composition and may explain some of the observed variations [47,48]. Future studies should investigate the potential of dietary interventions to modulate the intestinal microbiota and alleviate inflammatory symptoms. The bidirectional relationship between the host immune system and the intestinal microbiota necessitates further research to elucidate how specific microbial alterations impact health-related immune responses, and vice versa.

## 5. Conclusions

This study comprehensively characterized the fecal microbiota of Hainan black goats (Leizhou goats) stratified by peripheral WBC counts, revealing a significant association between the host’s hematological status and gut microbial community structure. High-throughput sequencing demonstrated substantial variations in both α-diversity and β-diversity among the Low, Middle, and High WBC groups, with PCoA confirming distinct separations in microbial community composition—notably, the High WBC group exhibited the highest α-diversity indices, indicating greater microbial richness and diversity.

The gut microbiota was predominantly composed of the phyla *Bacillota* (*Firmicutes*), *Bacteroidota*, and *Actinobacteriota*. Critically, we identified key microbial taxa strongly correlated with WBC counts, including the phyla *Spirochaetota* and *Fibrobacterota*, and genera such as *Ruminococcus*, *Treponema*, *[Eubacterium]_siraeum_group*, *Monoglobus*, and *norank_o_RF39*. Spearman’s correlation analysis further underscored significant relationships between these genera and major hematological parameters (WBC count, LYM%, MID%), with *unclassified_f_Oscillospiraceae* and *unclassified_c_Clostridia* showing strong positive correlations with WBC levels—highlighting their potential role as microbiota-mediated immune regulation markers.

These findings establish that gut microbiota composition is closely linked to goat hematological status, providing a foundational basis for future research into microbiota-driven immune regulation in this breed. While the study has limitations (e.g., limited sample size, fecal samples not fully reflecting intestinal microbiota [49]), it offers critical insights for developing microbiota-targeted health assessment and preventive strategies in goats. Future investigations should validate these associations with larger, temporally / spatially extended samples and explore the causal mechanisms underlying the gut microbiota-immune system axis.

## Figures and Tables

**Table 1:** Composition and nutrient levels of the experimental basal diet (dry matter basis).

Item	Parameter	Content (g·kg^−1^)
Feed ingredients	Corn	300.00
	Peanut hay	375.00
	Soybean meal	60.00
	Distillers’ dried grains with solubles	40.00
	Wheat bran	50.00
	Soybean hulls	130.00
	CaHPO_4_/Limestone	5.00
	Premix ^1^	40.00
	Total	1000.00
Nutritional components ^2^	Dry matter	889.20
	Crude protein	142.50
	Ether extract	32.30
	Ash	75.10
	Neutral detergent fiber	385.20
	Acid detergent fiber	210.40
	Calcium	6.80
	Phosphorus	4.20
	Metabolizable energy (MJ/kg) ^3^	10.22

^1^ Per kg of premix contains: Vitamin A, 150,000 IU; Vitamin D_3_, 50,000 IU; Vitamin E, 500 IU; Vitamin B_1_, 200 IU; FeSO_4_·H_2_O, 1800 mg; MnO, 1500 mg; ZnO, 1000 mg; KI, 10.0 mg; Na_2_SeO_3_, 3 mg; CoSO_4_, 5 mg; CaCO_3_, 100 g; Phosphorus, 3 g; NaCl, 100 g. ^2^ All nutrient compositions were determined experimentally [34]. All values, except for dry matter, are expressed on a dry matter basis. The concentrate-to-forage ratio is approximately 50.5:49.5. ^3^ The metabolizable energy value was calculated.

**Table 2:** Hematological parameters of goats categorized by WBC levels.

Parameter	Lower Group	Middle Group	High Group	*p*-Value
WBC(10^9^/L)	8.65 ± 1.42 ^a,b^	17.48 ± 1.51 ^a^	23.28 ± 4.21 ^b^	<0.001
LYM% (%)	0.09 ± 0.03	0.13 ± 0.07	0.05 ± 0.05	0.083
MCV (fL)	26.27 ± 3.43	25.93 ± 2.65	24.50 ± 2.31	0.324
MID% (%)	2.93 ± 0.52	3.50 ± 1.58	2.26 ± 0.75	0.103
HCT (%)	39.49 ± 9.86	36.57 ± 4.76	38.08 ± 9.99	0.829
RBC (10^12^/L)	14.86 ± 2.01	14.10 ± 0.93	15.44 ± 3.06	0.212
MCHC (g/L)	378.06 ± 57.39	372.17 ± 18.56	411.50 ± 64.01	0.218
GRAN% (%)	96.89 ± 0.45	96.37 ± 1.28	97.69 ± 0.80	0.087
GRAN# (10^9^/L)	8.44 ± 1.82 ^a,b^	16.90 ± 1.36 ^a^	22.09 ± 4.85 ^b^	<0.001

1. Abbreviations: WBC, white blood cell count; LYM%, lymphocyte percentage; MCV, mean corpuscular volume; MID%, mid-cell percentage (includes eosinophils, basophils, and monocytes); HCT, hematocrit; RBC, red blood cell count; MCHC, mean corpuscular hemoglobin concentration; GRAN%, granulocyte percentage; GRAN#, absolute granulocyte count. 2. Data are presented as mean ± SD. 3. Different superscript letters (e.g., ‘a’, ‘b’) within a row indicate statistically significant differences (*p* < 0.05) between groups, as determined by Kruskal–Wallis test with Bonferroni post hoc test.

## Data Availability

The original contributions presented in this study are included in the article/Supplementary Material. Further inquiries can be directed to the corresponding authors.

## Supplementary Materials

The following supporting information can be downloaded at: https://www.mdpi.com/article/10.3390/microorganisms14010259/s1, Table S1: Microbial community composition at the genus level.
